# Reply to Amaç, B. Comment on “Sklifasovskaya et al. Hypertension and Diabetes Cooperatively Drive HSP90 Activation, HSP70 Suppression, and Left Ventricular Interstitial Expansion: Relevance to Maladaptive Myocardial Remodeling. *Pathophysiology* 2026, *33*, 19”

**DOI:** 10.3390/pathophysiology33030047

**Published:** 2026-07-08

**Authors:** Anastasia P. Sklifasovskaya, Mikhail L. Blagonravov, Madina M. Azova, Sergey V. Kurevlev, Vyacheslav A. Goryachev, Sergey P. Syatkin, Tatyana Yu. Zotova, Daniil Yu. Prokofiev

**Affiliations:** 1 Institute of Medicine, RUDN University, 6 Miklukho-Maklaya St., Moscow 117198, Russia; azova-mm@rudn.ru (M.M.A.); kurevlev-sv@rudn.ru (S.V.K.); goryachev-va@rudn.ru (V.A.G.); syatkin-sp@rudn.ru (S.P.S.); zotova-tyu@rudn.ru (T.Y.Z.); prokofyev-dyu@rudn.ru (D.Y.P.); 2 Moscow Botkin Multidisciplinary Clinical and Scientific Center, Moscow 125284, Russia

We thank the commentators for their thoughtful and constructive review of our work [1]. We appreciate the opportunity to clarify several methodological points, which we believe will help readers interpret our findings more accurately. Below we respond to each concern in turn.

## 1. Comment 1: Lack of In Vivo Cardiac Functional Assessment

We confirm that our study was designed to evaluate molecular and histological endpoints under ethically approved terminal protocols (Approval No. 26, RUDN Institute of Medicine). This design choice was deliberate and aligns with established approaches for the initial mechanistic characterization of stress-response pathways.

We explicitly stated this limitation in the original manuscript (Discussion Section, “This study has several limitations”) [2] and framed our conclusions accordingly: the observed HSP90/HSP70 imbalance and interstitial expansion represent molecular correlates of maladaptive remodeling, not direct functional evidence. This interpretive boundary is standard in hypothesis-generating mechanistic studies and does not diminish the validity of the reported associations.

Future work incorporating echocardiography, pressure–volume loops, or biomarker profiling will be essential to establish causal links to cardiac performance. However, the present data provide a robust foundation for such investigations by identifying specific chaperone dysregulation patterns under comorbid stress.

## 2. Comment 2: Severity of the Streptozotocin-Induced Diabetes Model

The STZ-induced model (65 mg/kg, single i.p.) was selected to replicate insulin-deficient, uncontrolled diabetes, a clinically relevant phenotype for studying extreme cardiometabolic stress. The documented hyperglycemia, ketonuria, and weight loss (Table 1) confirm model fidelity and are consistent with established protocols in the field [2].

Importantly, several patterns in our data support a synergistic interaction between AH and DM, rather than a simple additive effect of hyperglycemia:

1. Non-linear HSP90 dynamics: In isolated DM, HSP90 protein increased ~4-fold; in AH+DM, and protein elevation was ~3.7-fold despite a 25.9-fold mRNA increase. This dissociation suggests comorbidity-specific post-transcriptional regulation not observed in isolated DM.
2. Divergent HSP70 regulation: HSP70 protein remained at control levels in isolated DM (19.50% vs. 19.70%) but was significantly suppressed in AH+DM (14.71%, *p* ≤ 0.05), despite comparable glycemic burden. This pattern is inconsistent with a glucose-driven effect alone.
3. Structural outcomes: Stromal volume fraction peaked in AH+DM (9.43%), exceeding values in isolated DM (7.18%) and AH (2.81–3.44%), indicating a supra-additive structural impact.

We acknowledge that model severity may amplify stress responses. However, this does not invalidate the observed comorbidity-specific patterns; rather, it underscores the clinical relevance of studying uncompensated states where synergistic damage is most pronounced.

## 3. Comment 3: RT-qPCR Methodology Inconsistencies

We clarify the following points regarding molecular methodology: TaqMan probe-based chemistry was employed exclusively during the initial assay development phase to independently validate primer specificity and confirm target amplification. Following successful validation, we transitioned exclusively to SYBR Green I chemistry (qPCRmix-HS SYBR, Eurogen) for all experimental data acquisition reported in the manuscript, as it proved optimal for our high-throughput workflow. The manuscript’s reference to TaqMan pertains solely to this independent validation stage and does not imply mixed or sequential optimization of chemistries. All quantitative measurements, post-run melt-curve analyses, and final statistical calculations were performed exclusively using SYBR Green I. Primer specificity was rigorously confirmed via melt-curve analysis, and amplification efficiencies were consistently ~1.98 across all targets. The reference to HSP60 mRNA was technical oversight. Actually, no HSP60 data were generated, analyzed, or reported in the present study. This sentence does not impact the methods, results, or conclusions concerning HSP70/HSP90.

We confirm that all RT-qPCR procedures adhered to MIQE guidelines. Primary amplification data, primer validation details, and amplification curves are available upon reasonable request.

## 4. Comment 4: Statistical Approach to Multiple Comparisons

Our statistical approach was pre-specified and internally consistent: each experimental group was compared a priori to the control group (WKY, normotensive, non-diabetic), reflecting our hypothesis-driven design focused on deviation from baseline. For these five planned comparisons per outcome variable, we applied the Holm–Bonferroni step-down procedure to control the family-wise error rate. All reported significant differences (*p* ≤ 0.05) remained significant after this adjustment (see Tables 2 and 3) [2].

Clarification on “no post hoc correction”: this statement indicated that we did not perform additional, exploratory pairwise comparisons (e.g., AH vs. DM, 38w vs. 57w AH) beyond the pre-specified control-based contrasts. No omnibus test (e.g., Kruskal–Wallis) was followed by post hoc testing, as our hypotheses were directional and focused on pathological deviation from norm.

This approach is appropriate for basic research with small sample sizes and focused hypotheses as our work was not conducted in the frame of pre-clinical studies. Effect sizes and confidence intervals are provided in Tables 2 and 3 to support interpretation beyond binary significance testing.

## 5. Concluding Remarks

We thank the commentators for their engagement with our work. The points raised have allowed us to clarify methodological details that reinforce the validity and interpretability of our findings.

Our study provides novel molecular and histological evidence that comorbid arterial hypertension and insulin-dependent diabetes drive synergistic HSP90 upregulation and HSP70 suppression in the left ventricular myocardium. These chaperone imbalances correlate with interstitial expansion and represent plausible mechanistic contributors to maladaptive remodeling in cardiometabolic disease.

While functional validation remains an important next step, the present data establish a foundation for targeted interventions aimed at restoring proteostatic balance under comorbid stress.
